# Reply to Lissing et al. Comment on “Ramai et al. Risk of Hepatocellular Carcinoma in Patients with Porphyria: A Systematic Review. *Cancers* 2022, *14*, 2947”

**DOI:** 10.3390/cancers15041187

**Published:** 2023-02-13

**Authors:** Daryl Ramai, Smit S. Deliwala, Saurabh Chandan, Janice Lester, Jameel Singh, Jayanta Samanta, Sara di Nunzio, Fabio Perversi, Francesca Cappellini, Aashni Shah, Michele Ghidini, Rodolfo Sacco, Antonio Facciorusso, Luca Giacomelli

**Affiliations:** 1 Division of Gastroenterology and Hepatology, University of Utah, Salt Lake City, UT 84112, USA; 2 Department of Internal Medicine, Hurley Medical Center, Michigan State University, Flint, MI 48503, USA; 3 Division of Gastroenterology & Hepatology, CHI Health Creighton University Medical Center, Omaha, NE 68131, USA; 4 Health Science Library, Long Island Jewish Medical Center, Northwell Health, New Hyde Park, NY 11040, USA; 5 Department of Internal Medicine, Mather Hospital, Northwell Health, Port Jefferson, NY 11777, USA; 6 Department of Gastroenterology, Postgraduate Institute of Medical Education and Research, Chandigarh, Sector 12, Chandigarh 160012, India; 7 Polistudium SRL, 20135 Milan, Italy; 8 Division of Medical Oncology, Fondazione IRCCS Ca’ Granda, Ospedale Maggiore Policlinico, 20122 Milan, Italy; 9 Gastroenterology Unit, Department of Surgical and Medical Sciences, University of Foggia, 71122 Foggia, Italy

We thank Dr. Lissing and colleagues for providing us with these helpful comments [1]. We are appreciative of your expertise and critical review of our work [2]. We have recognized that there were errors and will take the necessary steps to correct them.

After removing the overlapping articles, which in some cases were not very clear to us but following your assertions, we identified 13 articles, of which 3757 patients had porphyria (of any subtype) (Table 1). Overall, from this cohort, we identified 166 patients who developed cancer. We have also clearly laid out different types of porphyria and the number of cancer cases for each of those subtypes as per your recommendations; please see the table below.

While disease severity would be interesting to assess, however, this is difficult to extract from these studies. However, we agree that a large cohort study with this information, including age, would be important for future research efforts.

Again, thank you for your comments.

## Figures and Tables

**Table 1:** Characteristics of patients with porphyria.

Author/Year	Design	Location	Total Patients (*n*)	Total female *n* (%)	Age (Years), Mean ± SD	Age at Cancer Diagnosis (Years), Mean ± SD	α-Fetoprotein Levels	Porphyria Subtype with Cancer	Type of Cancer
Solis 1982 [3]	* Single-center	Spain	138 PCT	3 (2)	NR	64 ± 7	780 ng/mL (1) 1320 ng/mL (1) 2150 ng/mL (1) Positive (5) ND (2)	10 PCT	HCC (7) Unknown (3)
Salata 1985 [4]	Retrospective, single-center	Spain	83 PCT	6 (7.2)	57	60 ± 5	Elevated in 3 out of 9 HCC cases	13 PCT	HCC (13)
Siersema 1992 [5]	Prospective, single-center	Netherlands	38 PCT	13 (34)	48 ± 12	54 ± 4	None were elevated	5 PCT	HCC (5)
Kauppinen 1992 [6]	Retrospective, single-center	Finland	206 (184 AIP, 61 VP)	121 (58.7)	49 (Range 21–96)	NR	NR	6 AIP 1 VP	HCC (7)
Andant 2000 [7]	Prospective, single-center	France	650 (430 AIP, 136 VP, 84 HC)	347 (53)	41 ± 7	50 ± 10	>200 IU/mL (7)	5 AIP 1 VP 1 HC	HCC (7)
Fracanzani 2001 [8]	Case–control, single-center	Italy	53 PCT	2 (3.8)	56 ± 8	NR	>400 UI/mL (1)	18 PCT	HCC (18)
Gisbert 2004 [9]	Retrospective, Single-center	Spain	39 PCT	4 (10)	55 ± 16	69	Elevated (1)	1 PCT	HCC (1)
Cassiman 2008 [10]	Retrospective, single-center	Belgium	17 Sporadic PCT	7 (41)	43 ± 3	NR	NR	1 PCT	HCC (1)
Lang 2015 [11]	Questionnaire	Germany	122 (97 AIP, 20 VP, 4 HC, 1 ADDP)	NR	NR	NR	NR	1 AIP	HCC (1)
Baravelli 2019 [12]	Retrospective, multicenter	Norway	589 (243 sporadic PCT, 245 familial PCT, 101 unknown)	319 (52)	52 ± 13	NR	NR	Did not classify	HCC (6)
Baravelli 2017 [13]	Retrospective, population registry	Norway	251 (222 AIP, 21 VP, 8 HC)	151 (60.2)	Median (range) 53 (19–96)	NR	NR	8 AIP 1 VP	HCC (9)
Saberi 2020 [14]	Retrospective, multicenter	USA	327 (270 AIP, 19 HC, 38 VP)	266 (81)	32 ± 5	69 ± 5	<10 ng/mL (4)	4 AIP 1 VP	HCC (5)
Lissing 2022 [15]	Retrospective, population registry	Sweden	1244 (1063 AIP, 125 VP, 56 HC)	654 (53)	Median (range) 36 (19–53)	Median (range) 71 (53–89)	NR	81 AIP 1 VP 1 HCP	HCC (67), CC (3), unspecified (13)

* Study design (i.e., prospective, retrospective, etc.) unclear. AHP—Acute hepatic porphyrias, AIP—Acute intermittent porphyria, VP—Variegate porphyria, HC— Hereditary coproporphyria, PCT—Porphyria cutanea tarda, ADDP—δ-aminolaevulinic acid dehydratase-deficient porphyria, NR—Not reported, HCC—Hepatocellular carcinoma, and CC—Cholangiocarcinoma.

## Data Availability

Not applicable.
